# The Menopause and Its Disorders

**Published:** 1897-09

**Authors:** 


**The Menopause and its Disorders.** By A. D. Leith Napier, M.D. Pp. xv., 307. London: The Scientific Press, Limited. 1897.

If the author can demonstrate that there is a pathological condition at, or about the cessation of menstruation which influences the ordinary diseases to which women are liable or is itself a special disease, then we shall grant a reason for the production of this work, but we do not think that he can substantiate this position.

A great deal of space (75 pages) is taken up in considering theories of menstruation. These can be found in almost every work on Midwifery and Gynæcology, and might well be omitted from a work which from its title would lead one to expect that definite diseases were treated in a practical manner.

The chapter on the normal change of life is good, and it would have been well if the work had begun with it.

Under the heading of the disorders of the menopause the author correctly, in our opinion, puts down alcohol as the cause of a good many of the "nervous" symptoms complained of by patients. We believe that, independently of real disease, the abuse of alcohol produces more disturbance at the menopause than any error in diet.

The remainder of the work treats of conditions of ill-health which often exist at times other than at the menopause, and are dealt with fully in ordinary works on medicine. Also there is a good deal of space devoted to diseases which, although they often commence at the menopause, yet must not be considered incident to that time only.

